# Detection and Quantification of Extracellular Vesicles via FACS: Membrane Labeling Matters!

**DOI:** 10.3390/ijms21010291

**Published:** 2019-12-31

**Authors:** Fanny Ender, Piet Zamzow, Nikolas von Bubnoff, Frank Gieseler

**Affiliations:** Experimental Oncology, University Hospital and Medical School (UKSH), University of Luebeck, Ratzeburger Allee 160, 23538 Luebeck, Germany; Piet.Zamzow@uksh.de (P.Z.); Nikolas.vonBubnoff@uksh.de (N.v.B.); Frank.Gieseler@uksh.de (F.G.)

**Keywords:** extracellular vesicles, exosomes, ectosomes, flow cytometry, CFSE, threshold adjustment, COLO 357

## Abstract

The field of extracellular vesicle (EV) research is challenged by the lack of standardized protocols to identify and specifically distinguish between exosomes and ectosomes, which are released via exocytosis or plasma membrane shedding, respectively. Using sequential centrifugation, we separated EV subpopulations from supernatants of COLO 357 pancreas carcinoma cells based on size and mass. After 10,000× *g* centrifugation, we reconstituted high-speed (hs) EVs from the pellet, directly labeled them with the membrane dye carboxyfluorescein diacetate succinimidyl ester (CFSE), and performed flow cytometry based analysis. The aim was to optimize the conditions for EV labeling and detection and hence to obtain a maximum yield of intact hsEVs. We found that, for sufficient labeling of EVs, minimal temperature variations and short incubation times correlated with EV stability. Furthermore, threshold adjustment significantly improved the sensitivity of the flow cytometer for the detection of CFSE labeled hsEVs. When cells were CFSE labeled, we observed a transition of fluorescence onto EVs that were reconstituted from the pellet but not onto those that remained in the supernatant after hs centrifugation, suggesting the indirect labeling of EVs based on the way of biogenesis as a specific method for the distinction of exosomes and ectosomes. Protocol standardization is of major importance for the use of EVs as diagnostic markers in liquid biopsies.

## 1. Introduction

Before the development of high-resolution flow cytometers, small particles were simply excluded from the analysis of cellular preparations because they were seen as cellular debris or background noise. Today, we know that these particles are actively secreted as important mediators of intercellular communication. Furthermore, it has been shown that the so-called “extracellular vesicles” (EVs) play a decisive role in inflammatory processes as well as the development and the progression of malignant diseases [1,2]. However, the exact mechanisms by which EVs mediate physiological and pathological processes are incompletely understood. One of the reasons is that the research field is challenged by inconsistencies regarding EV isolation, purification, and characterization. Although EV subsets arise from different biological pathways (ectosomes: budding from the outer plasma membrane; exosomes: secreted from multivesicular bodies) [3] they overlap in size and yet no specific marker could be assigned to one or the other subset [4]. The most promising approach to separate ectosomes from exosomes is a combination of different methods for EV purification (e.g., sequential centrifugation, magnetic beads) and characterization (e.g., high-resolution flow cytometry, nanoparticle tracking analysis (NTA), electron microscopy). According to a worldwide survey in 2016, sequential centrifugation is the most common method used for primary EV separation and concentration [5]. We and others have used this method with small adaptations to successfully separate larger EVs from smaller EVs, showing differences in content and functionality of those subpopulations [6,7]. Enabling multi-parameter single particle analysis of EVs, high-resolution flow cytometry is considered as the most promising technique for ideal EV analysis. A high-resolution flow cytometer typically differs from a conventional machine by having: (1) high powered lasers with a smaller focused beam spot size, (2) a stable, slow velocity core stream with a small diameter, (3) smaller fluorescence/side scatter collection optical apertures and/or higher sensitivity detectors, and (4) larger forward scatter obscuration bars and higher sensitivity detectors [8].

Following the suggestion of the International Society for Extracellular Vesicles (ISEV), we chose an EV nomenclature that refers to the physical characteristics (size and mass) for the experimental preparations of this study [4]. Using both sequential centrifugation and high-resolution flow cytometry, we aim to contribute to the elaboration of a standardized protocol for EV isolation, differentiation, and quantification. We also want to highlight the importance of ectosomes, since they mirror the outer membrane of their cellular origin, such as tumor cells, and are thus promising candidates for use as biomarkers in patients’ body fluids (liquid biopsies).

## 2. Results

### 2.1. Purification and Detection and of High-Speed EVs

**Figure 1:**
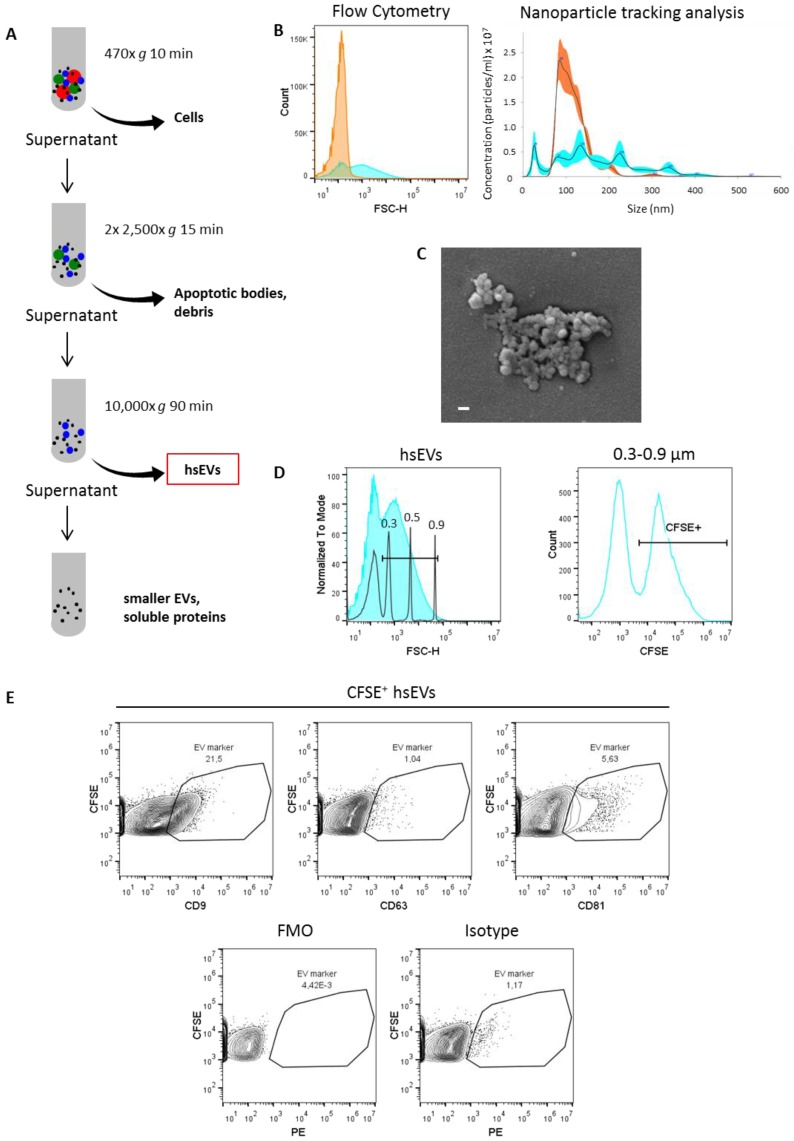
Identification and characterization of high-speed extracellular vesicles (EVs). (**A**) Purification of EV subsets using sequential centrifugation. (**B**) Analysis of EV size distribution by flow cytometry (left) and nanoparticle tracking analysis (NTA) (right). Orange histograms represent the supernatant, and cyan histograms represent the pellet after high-speed centrifugation. (**C**) Scanning electron microscopic image of high-speed (hs) EVs (pellet). Scale bar 100 nm. (**D**) Gating strategy for the identification of intact hsEVs by high-resolution flow cytometry. A gate representing the size range of interest (0.3–0.9 µm) was set according to known diameters of standard beads (left). Within the estimated size range of 0.3–0.9 µm, carboxyfluorescein diacetate succinimidyl ester (CFSE^+)^ events were identified as intact hsEVs (right). Samples were measured with a threshold of 0 for the FITC channel. (**E**) Flow cytometry analysis of tetraspanin markers CD9, CD63, and CD81 on hsEVs (upper panel), fluorescence minus one (FMO) control (lower panel left), and isotype control (lower panel right). Data shown are representative of three independent experiments.

For the purification of EV subpopulations, we used sequential centrifugation, as this method is reported to be the one most commonly used worldwide [5] (Figure 1A). Furthermore, we showed before that high-speed centrifugation separates EV subpopulations based not only on size and density but also on their coagulative capacity, since the amount of tissue factor (TF) activity was significantly higher on the surface of high-speed (hs) EVs after 10,000× *g* centrifugation when compared to smaller EVs that resided in the supernatant of malignant effusions from patients [7].

Using both flow cytometry (FC) (Figure 1B, left) and nanoparticle tracking analysis (NTA) (Figure 1B, right), we confirmed a selective enrichment of larger EVs in the pellet and smaller EVs in the supernatant after high-speed centrifugation. Of note, a sharp separation between EV populations based on size was not possible using sequential centrifugation due to overlapping sizes. Scanning electron microscopic images of COLO 357 cell line-derived hsEVs show small, round structures that we identified as EVs (Figure 1C). To ensure that we had intact EVs after running the centrifugation procedure, we fluorescently stained hsEVs using the cell permeable membrane dye carboxfluorescein diacetate succinimidyl erster (CFDA-SE). The intravesicular turnover of the non-fluorescent CFDA-SE to the fluorescent variant is dependent on the presence of active esterases and hence is indicative of an intact biological system [9]. To further confirm the lipid-bilayer structure specific of EVs, we assessed the presence of the tetraspanins CD9, CD63, and CD81 on the surface of hsEVs by FC. We found considerable amounts of CD9 and CD81 but not CD63 within independent isolations of hsEVs (Figure 1E, upper panel). Ensuring specificity of our results, we included a fluorescence minus one (FMO) (Figure 1E, left) as well as an isotype control (Figure 1E, right) in our analysis. Notably, CD9 and CD81 were not uniformly present within our preparations, as we detected these markers only on a subpopulation of CFSE^+^ hsEVs. We showed before that hsEVs are functionally active, as they are capable of inducing tumor cell migration in vitro [10]. After size calibration with megamix beads (size range of interest was 0.3–0.9 µm), we were able to quantify intact hsEVs based on the amount of CFSE^+^ events detected by FC (Figure 1D).

### 2.2. CFSE Staining Conditions Have to Be Adjusted for Sufficient Labeling of EVs

**Figure 2:**
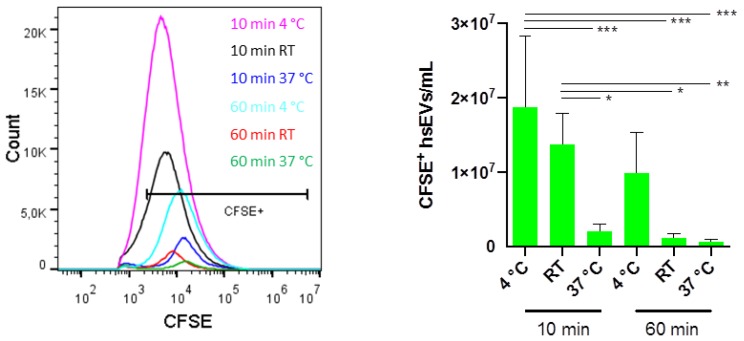
Optimization of CFSE-staining conditions for hsEVs. After low-speed centrifugation, the supernatant was harvested and stained with 40 µM carboxfluorescein diacetate succinimidyl erster (CFDA-SE) for 10 min or 60 min at 4 °C, room temperature (RT), or 37 °C. Samples were then centrifuged at 10,000× *g* for 90 min at 4 °C. Pellets were re-suspended in phosphate buffered saline (PBS). Quantification of CFSE^+^ hsEVs was done using high-resolution flow cytometry. A representative picture for the yield of intact hsEVs depending on the incubation condition is expressed by the histogram (left). Samples were measured with a threshold of 600 for the FITC channel. Bar graph shows the mean ± SD (*n* = 4) (right). Statistical analysis was done using two-way ANOVA and Tukey’s multiple comparison test. *p* values < 0.05 were considered statistically significant. * *p* < 0.05, ** *p* < 0.01, *** *p* < 0.001.

Performing membrane labeling with CFDA-SE at 37 °C ensures optimal conditions for enzyme activity and thus the turnover to the fluorescent variant CFSE. However, as the centrifugation protocol is always run at 4 °C, and variations in temperature are reported to negatively affect EV stability [4,11], we wondered if the labeling at 4 °C would not give a better yield of intact CFSE^+^ hsEVs. To get a complete picture, we compared 4 °C, room temperature (RT), as well as 37 °C. Additionally, we compared different incubation times for the labeling of hsEVs with the dye. Impressively, incubating hsEVs with 40 µM CFSE for 10 min at 4 °C or room temperature gave the best yield of intact CFSE^+^ hsEVs. Significantly lower amounts of CFSE^+^ hsEVs were detected after longer incubation times and/or at higher temperature, suggesting that longer incubation times and big temperature variations resulted in the degradation of hsEVs (Figure 2).

### 2.3. Protein-Containing Sample Buffers Generate Strong Background Signals

**Figure 3:**
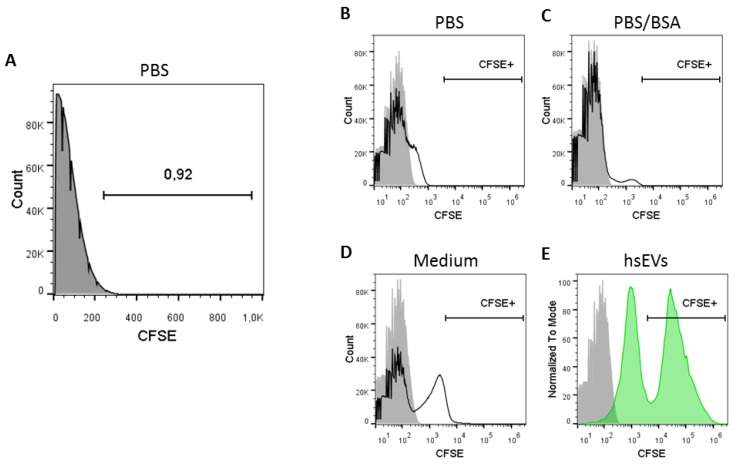
Selection of the appropriate sample buffer for hsEV reconstitution. (**A**) PBS without EVs and CFSE dye was measured to determine background noise. Events detected in the FITC channel are expressed in linear axis scaling format. Accordingly, a threshold of 250 in the FITC channel would exclude almost all events, since the gated area comprises less than 1%. We detected EV-independent signals over background noise when CFDA-SE was incubated in (**B**) PBS, (**C**) PBS/bovine serum albumin (BSA) or (**D**) cell culture medium. (**E**) The gate for detecting EV-dependent fluorescence signals in the FITC channel was set according to a CFSE-labeled hsEV sample. Samples were measured with a threshold of 0 for the FITC channel. Grey filled histogram = PBS without CFDA-SE; black line histogram = buffer with CFDA-SE; green filled histogram = CFSE-labeled hsEVs.

With 200 nm, the lower resolution limit of the Novocyte flow cytometer is in close vicinity to the expected size range of EVs. Therefore, a clear distinction between electronic noise and EV-derived signals is of major importance. Of note, measuring filtered phosphate buffered saline (PBS) (0.22 µm) alone resulted in the detection of 1.19 × 10^8^ ± 5.8 × 10^6^ events/mL (*n* = 4), which can be defined as background or electronic noise produced by the machine itself. Expressing the histogram axis in linear scaling format for software-based analysis, we were able to set the cut off for background signals at 250 (FITC channel) and thereby excluded 99% of the background noise (Figure 3A).

The situation got more complex when the membrane labeling dye CFSE was added to the sample buffer. Without any EV preparation (no enzymatic turnover), the addition of the dye to PBS sample buffer resulted in the detection of a weak CFSE^+^ signal in the fluorescent channel (Figure 3B). This EV-independent CFSE^+^ signal was stronger when PBS was supplemented with bovine serum albumin (BSA) (Figure 3C) or when complete cell culture medium was used (Figure 3D). Only the comparison with a CFSE-labeled hsEV preparation revealed that such a signal could be assigned to CFSE noise, as the EV-dependent CFSE signal was of a higher intensity (Figure 3E). In summary, to avoid any contaminating signal for the detection of CFSE-labeled hsEVs, we recommend using protein-free sample buffers such as pure PBS.

### 2.4. Threshold Adjustment Reduces Background Noise and Increases the Sensitivity for the Detection of CFSE-Labeled EVs

**Figure 4:**
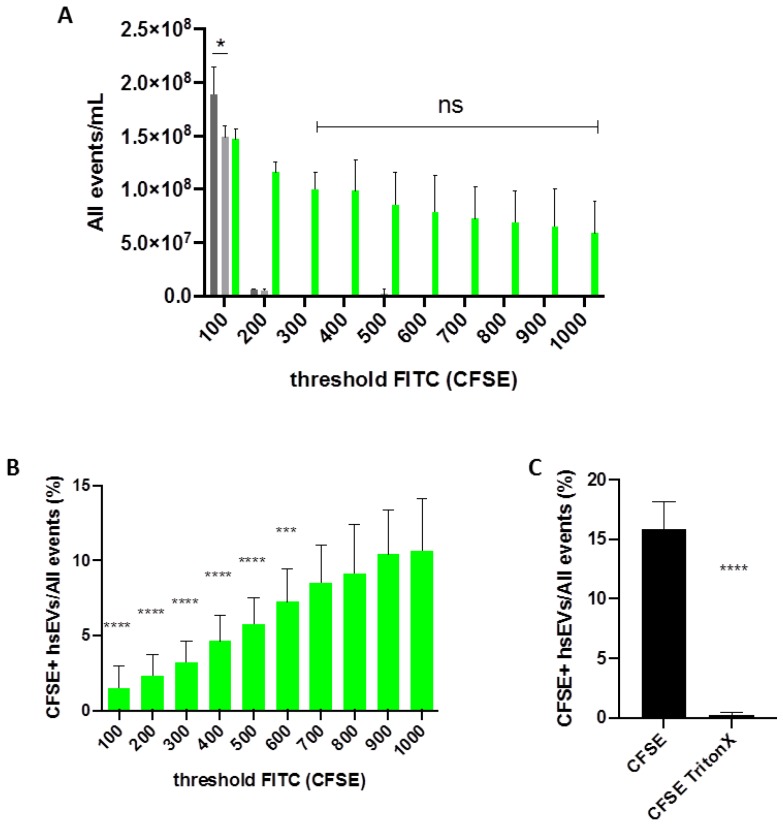
Impact of cytometer settings on the detection of hsEVs. (**A**) Shown is the total amount of events detected by the flow cytometer in the FITC channel and expressed as all events/mL. Bar graph shows mean ± SD (*n* = 4) for different threshold settings (100–1000). PBS control (dark grey), unlabeled hsEVs (light grey), CFSE-labeled hsEVs (green). For the comparison of PBS and unlabeled hsEVs, student’s *t*-test (two-tailed) was used. Statistical analysis of CFSE-labeled hsEVs (300–1000) was done using two-way ANOVA and Tukey’s multiple comparison test. *p* values < 0.05 were considered statistically significant. (**B**) Comparison of the relative amount of CFSE^+^ hsEVs within all events. Bar graph shows mean ± SD (*n* = 4) for different threshold settings (100–1000). Statistical analysis of CFSE-labeled hsEVs (threshold 100–1000) was done using two-way ANOVA and Tukey’s multiple comparison test. *p* values < 0.05 were considered statistically significant. Shown are the results of the multiple comparisons of 1000 to all the other values only. (**C**) HsEVs were CFSE-labeled and incubated with or without the detergent Triton X-100 (0.1%). Bar graph shows the impact of detergent treatment on the quantification of intact hsEVs. Samples were measured with a threshold of 1000 for the FITC channel representing the setting for the highest detection sensitivity. Statistical analysis was done using student’s *t*-test (two-tailed). *p* values < 0.05 were considered statistically significant. * *p* < 0.05, *** *p* < 0.01, **** *p* < 0.001, ns = not significant.

As mentioned before, detecting small EVs by FC is always a matter of working close to the detection limit. To get an overall impression of the sample contents, we first analyzed all events that were detected by the FC and displayed them in the FITC channel as the total amount of CFSE-positive and CFSE-negative signals (Figure 4A). When we included almost all signals by setting a low threshold for the FITC channel (100), we obtained counts for both unlabeled and CFSE-labeled hsEVs that were even below the counts from a PBS sample, suggesting that they disappeared within the background noise. Increasing the threshold for the FITC channel up to 300 excluded the background noise but kept detecting signals coming from the fluorescently labeled hsEV sample. After excluding background signals, the total amount of CFSE negative and CFSE-positive events detected was stable within a threshold range of 300 to 1000, since a two-way ANOVA revealed no statistical difference between those samples (Figure 4A, green bars). Subsequently, we analyzed the impact of threshold adjustment on the detection of CFSE^+^ hsEVs more in detail. When we set the threshold at 100, the machine detected only 1.5% CFSE^+^ events. In contrast, with a threshold set at 1000, we obtained 10.6% CFSE^+^ events (Figure 4B). This seven-fold increase in CFSE^+^ counts was highly significant, suggesting that threshold adjustment has a strong impact on the resolution of hsEV detection by FC. Additionally, we wanted to investigate how administration of detergent affects the detection of CFSE^+^ hsEVs. Treating CFSE-labeled hsEVs with Triton X-100 led to a highly significant decrease in CFSE^+^ hsEV detection, suggesting EV lysis as a consequence of treatment with the detergent and thus demonstrating once more that our EV preparations comprised intact vesicles (Figure 4C).

### 2.5. Tracking of Ectosome Shedding by CFSE Labeling of Their Cellular Origin

**Figure 5:**
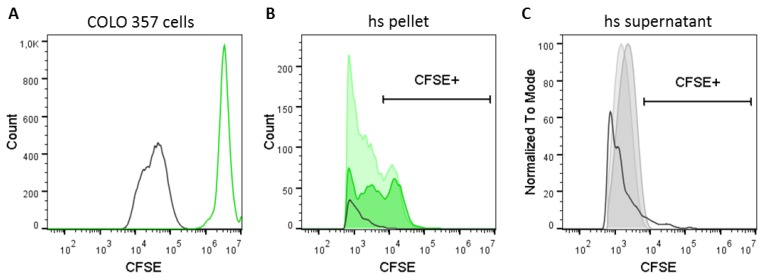
Tracking of ectosome shedding by CFSE-labeling of cells. COLO 357 cells were fluorescently labeled with CFSE and cultivated under standard cell culture conditions for 48 h. Cell culture supernatants were harvested 24 and 48 h after seeding, and cells were detached and collected from their surface 48 h after seeding. The presence of CFSE^+^ signals was determined (**A**) in cells after 48 h of cell culture as well as in (**B**) the pellet and (**C**) the supernatant after high-speed centrifugation (10,000× *g*) by flow cytometry. Samples were measured with a threshold of 600 for the FITC channel. Black line histogram = unstained control; green line histogram = CFSE-labeled COLO 357 cells; dark green filled histogram = hs pellet 24 h after seeding; light green filled histogram = hs pellet 48 h after seeding; dark grey filled histogram = supernatant 24 h after seeding; light grey filled histogram = hs supernatant 48 h after seeding. The CFSE^+^ gate was set according to the EV-dependent signal from a directly labeled hsEV preparation (see Figure 3E).

Cellular labeling with CFSE resulted in the detection of CFSE^+^ events with high fluorescence intensity (Figure 5A). However, as seen in Figure 3E and with the aid of the respective unstained controls, the cutoff for the identification of CFSE^+^ hsEVs was just below 10^4^ in the CFSE detection channel. Accordingly, we used a gate to detect fluorescence in EV subpopulations from the CFSE-labeled cell culture 24 h and 48 h after seeding. Indeed, we were able to detect a transition of fluorescence from cells to EVs over time but only in the pellet (Figure 5B) and not in the supernatant (Figure 5C) after high-speed centrifugation. In summary, membrane labeling of cells enables the tracking of ectosome shedding and hence might be a specific method to differentiate between ectosomes and exosomes based on the process of their biogenesis.

## 3. Discussion

In the presented study, we elaborated upon an optimized protocol for the detection and the quantification of EV subsets for cell culture supernatants. As suggested by the International Society for Extracellular Vesicles (ISEV), we chose an EV nomenclature that refers to the physical characteristics (size and mass) for the experimental preparations of this study [4]. Since membrane labeling of EVs resulted in higher numbers of EVs detected, EV quantities from unstained sample preparations are underrepresented. We could show that, for sufficient labeling of EVs, minimal temperature differences and short incubation times correlated with EV stability. During fluorescence triggering, threshold adjustment improved the EV detection sensitivity of our flow cytometer considerably, which can be considered as an important piece of information regarding data comparability between different laboratories. Furthermore, in light of the different biogenesis pathways of exosomes and ectosomes, fluorescent labeling of cells enables the tracking of ectosome shedding from the plasma membrane. Herewith, we provide a simple method allowing a specific distinction of ectosomes and exosomes based on their way of biogenesis. This method, however, is only applicable for cell culture systems and not for patient samples.

Here, we isolated and purified EV subsets from cell culture supernatants of COLO 357 cells by sequential centrifugation. Such protocols separate samples by size and mass and are commonly used in the field of EV research [5,7,12,13,14]. The final step of our EV purification protocol was a 10,000× *g* centrifugation resulting in the separation of EV fractions into the pellet and the supernatant according to their different sizes and masses. We focused on EVs reconstituted from the pellet after high-speed centrifugation that we designated “hsEVs”. Using flow cytometry and NTA, we could confirm that our protocol results in a selective enrichment of larger particles in the pellet. Electron microscopic images of hsEVs revealed round-shaped structures sticking together that we identified as EVs (Figure 1C). We showed before that an increased amount of phosphatidylserine (PS) on EVs is associated with a sticky phenotype [10]. The detectability of PS on the outer leaflet of ectosomes has been associated with their way of generation by budding from the plasma membrane of the cell of origin [15].

Ectosomes and exosomes are often described as particles with sizes of 100–1000 nm and 30−100 nm, respectively [2,16]. However, the applicability of techniques for a precise size determination strongly differs between currently available methods. Flow cytometry compares the scatter parameters of EVs with those of standard particles (e.g., polystyrene beads) of a known diameter. However, the intensity of FSC is not directly related to the particle size [17]. Additionally, light scattering also depends on the refractive index, the absorption coefficient, and the particle shape properties. Therefore, the characteristics of polystyrene beads significantly differ from those of cells and EVs [18]. In our study, we also refer to such beads for size calibration. For the identification of larger EVs (presumably ectosomes), we created a 0.3–0.9 µm size gate and analyzed the amount of intact hsEVs therein. We are aware that our results do not match this size range in equal measure, but it is applicable as a consistent parameter for the CFSE-labeling and -detection protocol optimization procedure. In comparison to flow cytometry, nanoparticle tracking analysis (NTA) is not influenced by the different refractive indices of size beads and biological material but uses the scattered light to detect a particle and tracks its motion as a function of time [19]. Using this method, we could show that EVs reconstituted from the pellet after high-speed centrifugation comprised a broad spectrum of particle sizes ranging from 80–400 nm, whereas a rather monodisperse EV population with a size of approximately 100 nm resided in the supernatant. This finding is in agreement with that of Cocucci and Meldolesi, who summarized the literature on similarities and differences between exosomes and ectosomes [3].

To demonstrate that purified hsEVs are intact biological objects, we used the membrane labeling dye CFSE, as the turnover of the non-fluorescent to the fluorescent variant is dependent on active esterases [20,21,22]. Furthermore, we detected considerable amounts of CD9 and CD81 on the surface of hsEVs (see Figure 1E), demonstrating the lipid-bilayer structure of EVs in general. Despite Western blotting being the most commonly used method for assessing the presence of proteins in total EV preparations, FC of membrane labeled intact EVs provides the advantage to evaluate the proportion of intact and surface marker positive events within the total preparation. Accordingly, we could show that the presence of these proteins is limited to a subpopulation of EVs. As sequential centrifugation separates EVs according to size and mass rather than based on the way of biogenesis, we cannot confidently ascribe the presence of CD9 and CD81 to exosomes or ectosomes. In light of our protocol optimization, the aim was to improve CFSE staining conditions (incubation time and temperature), ensuring hsEV stability during preparation and thus obtaining the highest yield of intact hsEVs. Enzymes of the human body typically have a temperature optimum of 37 °C characterized by high enzyme activity. However, the sequential centrifugation protocol was always run at 4 °C, which means that hsEVs have to withstand big temperature differences during the process of preparation. Therefore, we tested the impact of a CFSE incubation at 4 °C, room temperature, or 37 °C on the quantification of CFSE^+^ hsEVs by flow cytometry. Furthermore, we determined the influence of a 10 min and a 60 min CFSE incubation on hsEV counts. Indeed, keeping temperature variations at a low level during a short-term staining improves hsEV stability, since we obtained the highest amounts of CFSE^+^ hsEVs after a 10 min staining at 4 °C or at room temperature, which was significantly different from the other conditions tested. Although it is already known that temperature affects EV stability, researchers often only refer to storage conditions and suggest avoiding freeze-thaw cycles [4,11]. Only one study showed that longer incubation times improved EV staining but at the same time decreased EV concentration in a time- and a temperature-dependent manner [23].

After CFSE labeling, we performed sequential speed centrifugation, including high-speed centrifugation at 10,000× *g* for 90 min. Since we are interested in the identification and the characterization of biologically active EV sub-fractions from the pellet—in essence, ectosomes for reasons explained earlier—we had to find an appropriate sample buffer for reconstitution. Our demand on such a buffer was a minimal generation of background signals, alone and in combination with the dye, as well as a stabilizing impact on the hsEV preparations. Therefore, we tested PBS, PBS supplemented with BSA, as well as complete cell culture medium. It is known from cell-based flow cytometry that the addition of serum proteins maintains cell viability and maximizes fluorescence signal intensities generated by pH-sensitive fluorochromes [24]. In addition, as serum or serum proteins are known to decrease unspecific binding of antibodies [24], we considered the use of PBS/BSA as worthy in anticipation of future experiments dealing with antibody-based characterization of hsEV. Complete cell culture medium was tested as control. Surprisingly, without the presence of hsEVs at all, the addition of CFSE to the sample buffers tested resulted in the detection of a weak fluorescent-positive signal. Only in direct comparison to a CFSE-labeled hsEV sample could we confirm that this weak signal was EV-independent and could be referred to as CFSE noise (Figure 3). This phenomenon was observed before and was described as spontaneous hydrolysis of free dye causing unbound fluorescence. It can be reduced by the application of size exclusion chromatography [23,25]. In summary, we recommend the use of protein-free sample buffers, such as pure PBS, for reconstituting hsEVs from pellets after 10,000× *g* centrifugation to keep the level of potential contaminants low.

After optimization of CFSE incubation conditions and the selection of the appropriate sample buffer for hsEV reconstitution, we improved hsEV detection by adjusting the settings of the flow cytometer. As we found that unlabeled hsEVs mostly disappeared in the background noise due to the close vicinity to the detection limit of the machine, we decided first to follow the experience of Arraud et al., who described that triggering the detection by a fluorescence signal results in the detection of considerably more EVs than by the conventional approach based on light scatter triggering [26]. Second, we excluded all the background noise by setting the threshold on the fluorescence detection channel (FITC) to 250, which excluded 99% of the background noise coming from a PBS sample (see Figure 3A); further threshold increases from 300 to 1000 resulted in stable values. When we then determined the actual amount of CFSE^+^ hsEVs among all events, we found that increasing the threshold allowed the detection of even more CFSE^+^ events. In conclusion, the exclusion of events by threshold adjustment was compensated by an improvement of detection sensitivity. To the best of our knowledge, this has not been shown before, but we believe this is important for evaluating the comparability of data from different laboratories. Furthermore, improving the detection sensitivity may have an implication for sensing the expression of rare antigens in future experimental settings. According to the Minimal information for studies of extracellular vesicles 2018 guidelines [4], all machines and all techniques used for the identification and the characterization of EV preparations should be explained in detail. Based on our finding, threshold settings that are associated with fluorescence triggering should definitely be included in publications dealing with EV characterization.

In this study, we used CFDA-SE to label cells in vitro, as they are the source for EV generation. The non-fluorescent progenitor permeates biological membranes and is activated by esterases and covalently binds to free amines on the inner membrane leaflet as fluorescent variant (CFSE) [9,20,21,22]. Then, we cultivated CFSE-labeled COLO 357 cells and harvested cell free supernatants after 24 h and 48 h to determine the presence of CFSE^+^ EV in the pellet or the supernatant after high-speed centrifugation. We found CFSE^+^ EVs exclusively within a small sub-fraction of EVs that were reconstituted from the pellet after 10,000× *g* centrifugation, suggesting that fluorescence was transferred from cell to vesicle during the process of membrane shedding (Figure 5). We suggest that, by using this method, ectosomes generated from the outer cell membrane labeled by CFSE can be distinguished from exosomes generated inside the cell from multivesicular bodies not labeled by CFSE.

## 4. Material and Methods

### 4.1. Cell Culture

EV preparations used for this study were isolated from COLO 357 cell culture supernatants. This human cell line was once derived from a metastasis of a pancreatic adenocarcinoma and grew as an adhering monolayer with a cell doubling time of 21 h [27]. Cells were cultured under serum-free conditions using RPMI1640 (Lonza, Basel, Switzerland) supplemented with 10% panexin NTA (Pan-Biotech, Aidenbach, Germany), 1% penicillin streptomycin with glutamine (Gibco, Thermo Fisher Scientific, Waltham, MA, USA), and 1% sodium pyruvate (Biochrom, Berlin, Germany). Notably, we substituted FCS by panexin NTA to avoid contaminating our experimental preparation with FCS-derived EVs. Therefore, the concentration of FCS was diminished stepwise, and panexin NTA was increased over several weeks. After successful transition, COLO 357 cells were grown in a T75 flask covered by a total volume of 15 mL and incubated at 37 °C and 5% CO_2_. If a flask reached 80% confluency, the supernatant was collected and further prepared according to the protocol described below. Cell-free supernatants were kept at −80 °C until used. Supernatants were analyzed within 3 months after freezing to avoid EV degradation due to long-term storage. Cells were split using accutase (Sigma-Aldrich, St. Louis, MO, USA), and performing regular tests for detection of mycoplasma infections (Mycoalert, Lonza, Basel, Switzerland) revealed that COLO 357 cells were free of contamination.

### 4.2. CFSE Labeling of Cells and EVs (Direct Labeling)

For fluorescent labeling of cells and EV preparations, we used CFDA-SE, here and commonly referred to as CFSE (Cayman Chemical Company, Ann Arbor, MI, USA), a non-fluorescent progenitor of CFSE. CFDA-SE permeates biological membranes and gets activated by unspecific esterases inside the cell or the vesicle. Then, the fluorescent variant CFSE covalently binds to free amide groups of membrane proteins [21]. Membrane labeling was performed after low-speed and before high-speed centrifugation (as described below) of cell culture supernatants. We used CFSE at a concentration of 40 µM [10] and incubated the samples for either 10 or 60 min at 4 °C, room temperature, or 37 °C. To confirm the presence of intact EVs in our preparations, we administered the detergent Triton X-100 (Sigma-Aldrich, St. Louis, MI, USA) at a final concentration of 0.1% for 20 min at room temperature for EV lysis.

### 4.3. Antibody Labeling of EVs

To demonstrate the lipid-bilayer structure of hsEV by flow cytometry, the following antibodies against proteins of the tetraspanin family were used: anti-human CD9 Antibody (HI9a), anti-human CD63 Antibody (H5C6), and anti-human CD81 Antibody (5A6), all labeled with phycoerythrin (PE) and purchased from BioLegend (San Diego, CA, USA). Lacking specific target binding, we used an isotype control (mouse anti-human; IgG1κ) purchased from eBiosciences/Affymetrix (San Diego, CA, USA) in place of the primary antibody to determine the contribution of non-specific background to staining. Optimal antibody concentrations were titrated beforehand. Antibody labeling was performed for 20 min at 4 °C subsequent to CFSE labeling and just before high-speed centrifugation.

### 4.4. CFSE Labeling of EVs via Their Biogenesis (Indirect Labeling)

As ectosomes but not exosomes are generated by the outward budding of the plasma membrane, we investigated if membrane labeling of the cell of origin leads to the transition of fluorescence into one but not the other EV subpopulation over time. Therefore, 1.5 × 10^6^ COLO 357 cells were labeled with 40 µM CFSE for 10 min at 37 °C. Following a washing step, cells were re-suspended in a total volume of 5 mL cell culture medium and seeded into a T25 flask. Twenty-four hours and 48 h after seeding, we purified EVs from cell culture supernatant by sequential centrifugation (as described below) and analyzed the different fractions for the presence of CFSE^+^ EVs.

### 4.5. Sequential Centrifugation

In our study, EVs were enriched using the protocols previously published by Lacroix et al. [12] and Muralidharan-Chari et al. [13]. In short, samples were thawed at room temperature. For depletion of cells and large cell debris including most apoptotic bodies and large oncosomes, samples were spun twice at a low-speed centrifugation of 2500× *g* for 15 min (Cenrifuge 5430R, Eppendorf AG, Hamburg, Germany), and the supernatant was collected. Membrane labeling was done as described above. Subsequently, the labeled supernatant was spun at 10,000× *g* for 90 min (Cenrifuge 5430R, Eppendorf AG, Hamburg, Germany). The pellet was then collected and re-suspended in 0.22 µm filtered PBS (Figure 1A). In addition, PBS supplemented with bovine serum albumin (BSA) (Roche Diagnistics International, Rotkreuz, Switzerland) and complete cell culture medium was used as sample buffer to determine background noise or potential interactions with the membrane dye CFSE.

### 4.6. High-Resolution Flow Cytometry

For the analysis of hsEV preparations, we used a high-resolution Novocyte flow cytometer (ACEA Biosciences Inc., San Diego, CA, USA). The device was equipped with a blue laser (488 nm) and an auto sampler allowing the procession of 96-well plates. We loaded plates with 100 µL sample suspensions per well and set the stop condition to 25 µL per sample. To create a stable, slow velocity core stream recommended for the detection of EVs, the sample acquisition speed was adjusted to 10 µL per min, resulting in a core diameter of 6.5 µm. For the detection of larger cells, the speed was set to 64 µL per min and a corresponding core diameter of 16.6 µm. We used the forward scatted light to estimate the particle size within our preparations. In parallel, we compared the scatter parameters of our particles with those of standard particles of a known diameter. These Megamix Plus FSC beads (Biocytex, Marseille, France) comprised distinct populations (0.3, 0.5, and 0.9 µm of size), which allowed defining a size range of 0.3–0.9 µm for the detection of EVs. Within this size range, we quantified CFSE^+^ events as intact hsEVs. Threshold triggering was done on the fluorescence channel (FITC) to discriminate sample-derived signals from background noise and to evaluate the sensitivity of the detection system. Threshold settings ranged between 100 and 1000. Data generated by the Novocyte were analyzed using the NovoExpress software version 1.2.4 (ACEA Biosciences Inc., San Diego, CA, USA).

### 4.7. Nanoparticle Tracking Analysis

Nanoparticle tracking analysis (NTA) visualizes and measures small particles (10–1000 nm) in suspension based on the analysis of Brownian motion from a video sequence. Particles in the sample were visualized by the illumination with a laser beam. The scattered light of the particles was recorded with a light-sensitive camera, which was arranged at a 90° angle to the irradiation plane. The 90° arrangement allowed detection and tracking of the Brownian motion of vesicles 10 to 1000 nm in size. Using a special algorithm, particles were detected, and their path was registered. The size of each individually tracked particle was calculated, thus simultaneously allowing determination of their size distribution and concentration. For this study, we used an NS300 (Malvern Instruments Ltd., Malvern, UK) equipped with a 488 nm laser module.

### 4.8. Electron Microscopy

Isolated EVs intended for scanning electron microscopy (SEM) were fixed with glutaraldehyde at a final concentration of 2.5% in filtered PBS and stored at 4 °C until further preparation. After homogenization, 10–20 mL of each sample was placed onto a Thermanox coverslip (Thermo Fisher Scientific, Waltham, MA, USA) and allowed to settle for 90–120 min in a humid chamber to prevent drying. For dehydration, the samples were then placed into solutions with increasing acetone concentration (70–100%) and subsequently fully dried via critical point drying using CO_2_ to avoid shrinkage effects and loss of structure from air-drying. The dehydrated samples were sputtered with gold and analyzed with an EVO LS 15 scanning electron microscope (Carl Zeiss AG, Oberkochen, Germany).

### 4.9. Statistical Analysis

Statistical analysis was performed using GraphPad Prism version 8.2.1. (San Diego, CA, USA) Data were analyzed using two-tailed student’s *t*-test or two-way ANOVA (Tukey’s multiple comparison test) depending on the sample size. Values of *p* < 0.05 were considered statistically significant. All data are described as mean ± SD.

## 5. Conclusions and Outlook

In this paper, we describe several improvements and requirements to correctly characterize different EV sub-populations. Such discrimination is of potential clinical relevance, as ectosomes presenting antigens from their (tumor) cell of origin could be used as biomarkers in liquid biopsies. As we provide a method for the specific distinction of exosomes and ectosomes according to their biogenesis, further characterization of EVs regarding surface antigen expression or functionality has to be considered in future experiments.
